# Juvenile xanthogranuloma of larynx and trachea, case report of a life-threatening condition

**DOI:** 10.1177/00368504261440764

**Published:** 2026-07-27

**Authors:** Olivier Janjic, Arti Wakudkar, Victoria Salati, François Gorostidi

**Affiliations:** 1 Department of Oto-Rhino-Laryngology - Head and Neck Surgery, 27230 Geneva University Hospitals, Geneva, Switzerland; 2 Department of Clinical Pathology, 30635 Lausanne University Hospital (CHUV), Lausanne, Switzerland; 3 Department of Oto-Rhino-Laryngology - Head and Neck Surgery, 30635 Lausanne University Hospital (CHUV), Lausanne, Switzerland

**Keywords:** juvenile xanthogranuloma, larynx, trachea, pediatric airway, histiocytosis, case report

## Abstract

Juvenile xanthogranuloma (JXG) is a rare benign histiocytic disorder, typically affecting the skin of infants and young children. Extracutaneous manifestations are uncommon, and laryngeal involvement is exceedingly rare but potentially life-threatening. We report the case of a young girl with a one-year history of dysphonia and dyspnea. Laryngoscopy revealed a JXG of the left vocal fold which was endoscopically removed. The patient remains to this date symptom-free without recurrence. Only eleven cases of upper airway or tracheal JXG have been published so far. Therapeutic strategies range from tracheostomy to endoscopic excision. Our case underscores the risks of airway obstruction in this otherwise benign condition. It provides guidance for general management and highlights the importance of complete resection and close follow-up, given the notable risk of recurrence.

## Introduction

Juvenile xanthogranuloma (JXG) is a rare and benign histiocytic disorder most commonly seen in infants and young children. Typically presenting as cutaneous nodules, JXG usually involves the skin on the head, neck, and trunk. Although extracutaneous manifestations are uncommon, JXG lesions can possibly be localized at any site.^1^ Laryngeal location is exceedingly rare, with only a limited number of cases documented in medical literature and only one case involving the glottic space.^2^

Involvement of the larynx may result in progressive dysphonia, respiratory obstruction and stridor, often prompting evaluation by ENT specialists. Certain more advanced cases even necessitated urgent tracheostomies.^3,4^ Diagnosis is usually delayed, as laryngeal lesions in children are most commonly attributed to more frequent conditions, such as vocal fold nodules or polyps.^5^

We describe here the case of a young girl with a JXG of the larynx who exhibited a year-long history of progressive dysphonia and mild respiratory symptoms. She was successfully treated with endoscopic surgery.

This case highlights the importance of considering rare histiocytic disorders like JXG in pediatric patients with persistent laryngeal lesions, especially when initial therapy fails to resolve symptoms. The successful identification and surgical management of this patient’s JXG lesion provide valuable insight into the diagnosis and treatment of this rare pediatric laryngeal condition. Given the limited literature on its presentation and progression, we believe this case report can help guide clinicians in its assessment, management, and follow-up.

## Case report

**Figure 1.**
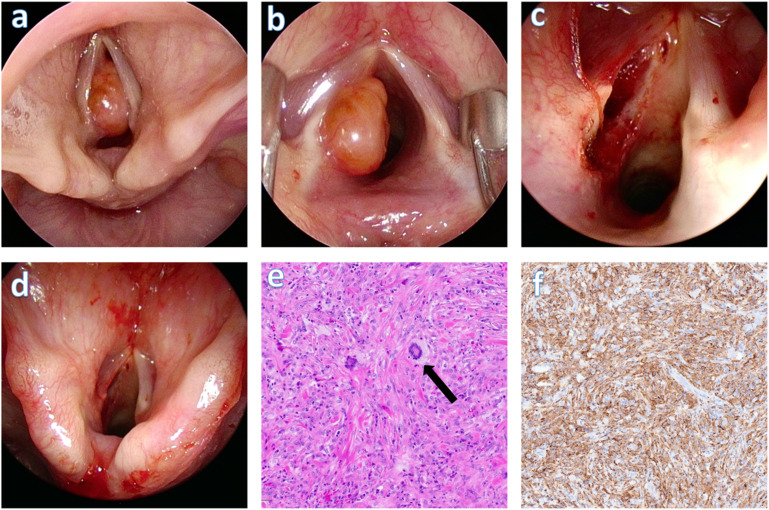
**a/b**: Endoscopic view revealing a multi-lobulated lesion on the posterior third of the left vocal fold. **c/d**: Larynx examination after surgical excision. **e**: Sheaths of foamy macrophages mixed with lympho-plasmocytic inflammatory infiltrate. **f**: Foamy macrophages positive for CD68 in immunohistochemistry. **Arrow**: Touton type giant cell.

A previously healthy young girl presented with a one-year history of progressive dysphonia, marked by a hoarse, strained voice that failed to improve with speech therapy. While she did not experience baseline dyspnea, she reported abnormally loud breathing even with mild exertion. On examination, her voice was consistently hoarse and strained, with frequent aphonic episodes but no stridor. Video-laryngoscopy revealed an exophytic, multi-lobulated lesion on the posterior third of the left vocal fold, extending to the arytenoid. The lesion was smooth and soft, occupying approximately two-thirds of the glottic opening (Figure 1, images **a** and **b**). The left vocal fold movement was not impaired, but the lesion prevented full glottic closure during phonation. The patient was admitted on October 2024 at the university hospital of Lausanne (CHUV), Switzerland. After obtaining informed consent, we proceeded with an endoscopic procedure for diagnosis and treatment. We did not perform prior imaging as there was no significant airway obstruction. Suspension microlaryngoscopy was performed, and the lesion was excised using a CO_2_ ultrapulse laser (1 W, 125 mJ). An incision was made along the lower surface of the left vocal fold mucosa, followed by careful dissection along the lateral border of the lesion into the subglottic space, preserving the vocal ligament and surrounding healthy tissue (Figure 1, images **c** and **d**). Hemostasis was achieved with bipolar cautery and adrenaline-soaked gauze. The procedure was well tolerated, and the patient remained clinically stable postoperatively. She was discharged the next day with corticosteroids for five days (Prednisone, 1 mg/kg, once a day) and proton pump inhibitors for one month (Pantoprazole, 20 mg, once a day). One week later, she developed a large granuloma at the resection site, requiring semi-urgent endoscopic removal. After six months of clinical follow-up, she remains symptom-free, with no signs of recurrence. A full dermatological assessment revealed no other lesions. Written informed consent has been obtained from the parents of the patient to publish this paper. All patient details have been de-identified. The reporting of this study conforms to CARE guidelines.^6^

## Pathology

Histological sections showed laryngeal nodule lined by pseudostratified columnar epithelium without atypia. The underlying lamina propria is infiltrated by sheets of foamy macrophages intermixed with lymphoplasmacytic inflammatory infiltrate. Scattered Touton-type giant cells were also observed. This proliferation infiltrated the striated muscles in depth and arrived at the surgical cut margin. In immunohistochemical analysis, these cells were diffusely positive for CD68-PGM1 and CD31 and negative for the other markers analyzed (S100, SOX10, CK AE1-AE3, smooth muscle actin, Desmin, and ERG). Histologic images are shown in Figure 1.

## Discussion

JXG belongs to the large and heterogeneous group of *histiocytosis*. It is subclassified in the C group, which includes the non-Langerhans cell histiocytosis of skin and mucosa. The exact cause of JXG remains for the moment unknown but is thought to be a reaction to an unknown stimulus. The immunohistochemical phenotype is usually CD68+, CD163+, FXIIIa+, CD1a–.^1^

The classic histology of JXG reveals a dermal infiltrate of spindle cells, mononuclear cells and multinucleated giant cells. Those latter may or may not display the characteristics of Touton giant cells (ring or wreath of nuclei surrounded by a foamy cytoplasm). The presence of lymphocytes, eosinophils, neutrophils, and mast cells can also be observed. Early JXG shows only histiocytes or spindle-shaped fibrohistiocytic cells minimally lipidized. More mature JXG usually shows foamy, lipid-laden, vacuolated mononuclear cells and Touton giant cells.^7^

JXG is a benign condition that can develop at any pediatric age but happens more in children than young adults (in majority in the first year of life). The classic appearance is a single nodule (measuring more than 2 cm) or multiple papules that are reddish to yellow-brown in color. The differential diagnosis includes mastocytoma, Spitz naevus, and, most importantly, other C-group histiocytoses. These entities often show considerable overlap with JXG, making diagnosis particularly challenging. Although JXG is the most common lesion within the C-group and is primarily distinguished by clinical parameters such as patient age, presentation, and disease course, its histological and immunohistochemical features are nearly indistinguishable from those of related conditions.^8^ On this matter, Emile et al. adds that histopathology and phenotype of extracutaneous or disseminated JXG do not differ from Erdheim-Chester disease (ECD), which is a histiocytosis of the intermediate group associated with a poor prognosis. While ECD normally shows a typical clinical presentation (e.g. skeletal involvement) distinguishing it from JXG, it does not always do. They thus recommend performing molecular analysis of these cases, and to consider as ECD all extracutaneous or disseminated JXG with gain-of-function mutation of BRAF, NRAS, KRAS,or MAP2K1.^9^

The prognosis of JXG is good as lesions are expected to regress spontaneously over a few years. When it can theoretically develop in any site, it mainly involves the head, neck and upper trunk skin regions, as well illustrated by the case reported by Baran et al.^10^ Depending on the extent and location of the lesions, JXG can have important impact on an individual’s quality of life. The eye involvement is a well-documented situation occurring in less than 1% of all JXG cases. It can lead to impairing complications such as anterior hemorrhage, glaucoma or uveitis.^7^

Our case shows the inherent dangerousness of a benign lesion developing at a critical anatomical area, namely the narrow airway space of the larynx. Although this remains an exceptional situation, a small number of similar cases have been reported in the literature. We revised the literature and found 11 other cases involving the upper respiratory airways and the trachea. Their characteristics are summarized in Table 1. The mean age of affected patients was 3.8 years, with cases ranging from 19 weeks to 8 years. The majority presented with stridor, respiratory distress, cough, or dysphonia, with symptom duration varying from a few days to several years. The lesions ranged in size from 1 to 3 cm and were located at various sites within the larynx but mainly at the subglottis and proximal trachea. No extralaryngeal lesions were identified. De Soccio et al. reported additional cutaneous lesions that were ultimately diagnosed as cutaneous mastocytosis.^11^ Comprehensive evaluation frequently included CT scans or MRI, along with additional investigations aimed at identifying concomitant lesions, such as abdominal ultrasound or slit-lamp examination. The tumors commonly present as yellowish or reddish fleshy masses, often sessile, smooth-surfaced that may appear cystic or polypoid in nature. The majority of cases were treated with excision using different techniques. Some authors specified using some adjuvant medical treatments such as steroid, Mitomycin-C of Vinblastine. Relapses were reported in 3 cases, occurring after 6 weeks, 5 months and one year.^2,12,13^

**Table 1.** Summary of laryngeal and tracheal xanthogranuloma cases.

Reference	Age	Sex	Initial symptoms	Symptoms duration	Location	Assessment	Treatment	Follow-up	Outcome
Benjamin, 1995^3^	19 w	F	Stridor	6 w	Bilateral aryepiglottic folds	Chest X-ray, Abdominal US	Tracheotomy, Observation	11 m	Spontaneous regression and decannulation
Thevasagayam, 2000^4^	3 y	F	Airway obstruction, Stridor	9 m	Posterior subglottis	MRI	Emergency tracheostomy, Observation	28 m	Spontaneous regression and decannulation
Sahhar, 2003^14^	18 m	F	Stridor, Dyspnea	2 w	Subglottis	CT scan	Laser resection	NA	Favorable
Naiman, 2004^12^	8 y	M	Airway obstruction, Stridor	5 m	Trachea (posterior wall, submucosal)	CT scan, MRI	- Laser resection	2.5 y	- Relapse after 1y
							- Relapse: surgical resection (external approach)		- Favorable
Li, 2006^15^	7 y	M	Cough, Stridor, Dyspnea	1 m	Proximal trachea	CT scan, Chest X-ray, Abdominal US	Tracheal resection, Safety tracheostomy	2 m	Not decannulated
Cevit^16^	8 y	M	Cough, Stridor	1 m	Proximal trachea	CT scan	“Therapeutic rigid bronchoscopy”	8 m	Favorable
Somorai, 2007^17^	5 m	M	Stridor, Respiratory distress	1 w	Subglottis	Slit-lamp exam, Bone marrow biopsy, CT scan, MRI, Bone scan, Chromosome analysis	- Debulking (Rigid bronchoscopy), Inhaled steroids	17 m	- Tumor progression after 4 m
							- Tumor progression: laser excision		- Favorable
Wang, 2010^18^	9 m	M	Stridor, Dyspnea	6 w	Left aryepiglottic fold	CT scan, Laryngeal US	“Surgical resection”	NA	NA
Kawamoto, 2013^2^	3 y	F	Hoarseness, Stridor	2 w	Right vocal fold to subglottic region	CT scan	- Resection (LMS)	7 m	- Relapse after 6 w
							- Relapse: Laser resection		- Favorable
De Soccio, 2020^11^	4 y	M	Cough	NA	Posterior wall of the oropharynx	“Extensive clinical workup”	“Resection”	11 m	Favorable
Verma, 2022^13^	4 y	F	Stridor, Respiratory distress	Acute onset	Subglottic	CT scan	- Laser resection, Mitomycin C, Steroids	5 m	- Relapse after 5 m
							- Relapse: Steroids and Vinblastine		- Partial response
Our case	8 y	F	Dysphonia	1 y	Left vocal fold	Ophthalmologic exam	- Laser resection	6 m	- Granuloma formation after 1 w
							- Granuloma formation: Laser resection		- Favorable

Abbreviations: CT = Computed Tomography, MRI = Magnetic Resonance Imaging, US = Ultrasound, NA = Not Available, w = week-s, m = months, y = year-s, LMS = laryngomicrosurgery.

Based on the literature, we recommend conducting a dermatologic and an ophthalmologic examination, due to the potential for ocular involvement and its associated complications. Several authors have reported an association between JXG, neurofibromatosis type 1, and juvenile chronic myelogenous leukemia. Consideration may be given to extended hematologic screening in relevant cases.^19^ As noted above, specific identification of histiocytic lesions can be challenging.

In addition to the histiocytic lineage markers employed in our case, the use of CD163, factor XIIIa and CD1a may be useful to further support diagnostic characterization in similar cases. A complete pathology work-up may also include molecular analysis for BRAF, ALK, and NTRK alterations, particularly given the availability of targeted therapies for NTRK fusion–associated diseases.^9,20–22^ These immunohistochemical and molecular analyses were not performed in the present case, as the diagnosis was considered sufficiently supported by the clinical presentation and the histological and immunohistochemical findings. Moreover, the lesion demonstrated a favorable clinical course following complete surgical excision. Although we performed no imaging, we strongly recommend its use to accurately assess the extent of the lesion. Notably, our intraoperative findings revealed an unexpected deep extension into the larynx at the glottic level, which had not been clearly identifiable preoperatively.

The management of upper airway JXG through tracheostomy and observation was chosen in 2 cases and seems a reasonable approach, given that JXG is known to regress spontaneously and that tracheostomy offers a reliable method for ensuring airway patency.^3,4^ This strategy mitigates the immediate risks associated with surgical intervention and anesthesia. However, considering the potential morbidity associated with prolonged tracheostomy, alongside the substantial advancements in laryngotracheal surgery and postoperative care in recent years, tracheostomy should be regarded as a last-resort option in the treatment of tracheal and upper airway JXG. In accordance with the recommendations of Classen et al., we propose endoscopic resection as the primary therapeutic approach, provided that the procedure does not create an excessive risk to the patient.^23^ Our small review suggests that the choice of resection technique does not seem to influence clinical outcomes. Among the ten cases managed with resection, only three exhibited a directly favorable postoperative course.^11,14,16^ Three cases experienced recurrence, and one case of partial resection resulted in disease progression.^2,12,13,17^ Including our case, where a granuloma developed shortly after resection, five cases in total – representing half of all the cases - required a secondary endoscopic intervention. These findings underscore the importance of achieving the most extensive and complete excision possible while ensuring close follow-up. We thus recommend a rigorous follow-up, particularly within the first three months, after which surveillance may be progressively spaced. Furthermore, in the case described by Naiman et al., recurrence was observed after one year, leading the authors to advocate for lifelong follow-up.^12^ While such an approach may be considered excessive, maintaining a high level of clinical vigilance remains essential to promptly identify and manage potential recurrences.

## Conclusion

This case highlights the rare but potentially life-threatening presentation of juvenile xanthogranuloma in the upper airway. We believe that every effort should be made to treat it conservatively and avoid tracheotomy at all costs, considering the high morbidity associated with it. Despite its benign nature, the anatomical location can pose significant risks, necessitating timely diagnosis, appropriate preoperative imaging and tailored intervention. Based on the available literature and our experience, secondary prompt endoscopic intervention is frequently required, supporting the need for close follow-up.

## Data Availability

Data supporting this study are included within the article.*
